# Global Surveillance, National Surveillance, and SARS

**DOI:** 10.3201/eid1002.031038

**Published:** 2004-02

**Authors:** David L. Heymann, Guénaël Rodier

**Affiliations:** * World Health Organization, Geneva, Switzerland

**Figure:**
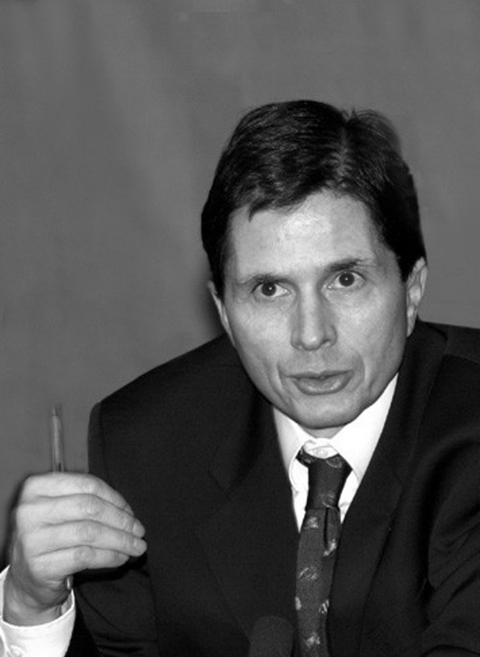
**David L. Heymann**  Executive Director, Communicable Diseases, World Health Organization, Geneva, Switzerland

The international response to the severe acute respiratory syndrome (SARS) outbreak, from March to July 2003, tested the assumption that a new and emerging infection–one that had not yet demonstrated its full epidemiologic potential but was spreading from person to person and continent to continent–could be prevented from becoming endemic. Within 4 months after the first global alert about the new disease, all known chains of transmission had been interrupted in an outbreak that affected 27 countries on all continents. Most public health experts and scientists believe that the question of whether SARS has become endemic can only be answered after at least 12 months of postoutbreak surveillance. The SARS experience, however, made one lesson clear early in its course: inadequate surveillance and response capacity in a single country can endanger national populations and the public health security of the entire world. As long as national capacities are weak, international mechanisms for outbreak alert and response will be needed as a global safety net that protects other countries when one nation’s surveillance and response systems fail.

During the last decade of the 20th century, several outbreaks, including cholera in Latin America, pneumonic plague in India, and Ebola hemorrhagic fever in the Democratic Republic of the Congo, caused great international concern (*1*–*3*). These events demonstrated the consequences that delayed national recognition and response to outbreaks could have: illness and death of national populations including health workers, potential spread to other countries, and significant disruptions of travel and trade. These outbreaks also emphasized the need for a global surveillance and response mechanism. The Global Outbreak Alert and Response Network (GOARN), set up in 1997 and formalized in 2000, was one major response to this need (World Health Organization [WHO], unpub. data and *4*). Though the network, which now has 120 partners throughout the world, currently identifies and responds to more than 50 outbreaks in developing countries each year, the SARS outbreak was the first time that GOARN identified and responded to an outbreak that was rapidly spreading internationally.

One of the partners in GOARN is the WHO Global Influenza Surveillance Network, which was established in 1947 to guide the annual composition of vaccines and provide an early alert to variants that might signal the start of a pandemic of rapidly evolving influenza viruses. This network was placed on alert in late November, when the Canadian Global Public Health Intelligence Network (GPHIN), also a partner in GOARN, picked up media reports of an influenza outbreak in mainland China (*5*). Simultaneously, another GOARN partner, the U.S. Global Emerging Infections Surveillance and Response System (GEIS), became aware of similar reports about a severe outbreak, with influenza B the suspected cause, in Beijing and Guangzhou. As GOARN continued to receive reports about influenza outbreaks in China, WHO requested information from Chinese authorities on December 5 and 11. On December 12, WHO received a detailed report on data collected at Chinese influenza surveillance sites, indicating that investigation of 23 influenza virus isolates had confirmed type B strains in all but one and that the number of cases was consistent with the seasonal pattern in previous years. The information was reassuring and an indication that the influenza surveillance system was working well.

Although information is incomplete, retrospective case identification by Chinese and GOARN epidemiologists since May 2003 suggests that two respiratory disease outbreaks occurred in Guangdong Province in late November 2002: influenza and what now appears to have been a first wave of SARS cases—an atypical pneumonia that was characterized by small, seemingly unrelated clusters of cases scattered over several municipalities in Guangdong, with low-level transmission to healthcare workers (*6*). This first wave of atypical pneumonia appears to have continued until a second wave of disease with amplified transmission to health workers began occurring during the first 10 days of February (WHO, unpub. data). On February 10, 2003, the WHO office in Beijing received an email message describing an infectious disease in Guangdong Province said to have caused more than 100 deaths. On February 11, the Guangzhou Bureau of Health reported to the press more than 100 cases of an infectious atypical pneumonia outbreak that had been spreading in the city for more than 1 month. That same day, the Chinese Ministry of Health officially reported to WHO 300 cases and 5 deaths in an outbreak of acute respiratory syndrome, and the next day reported that the outbreak dated back to November 16, 2002, that influenza virus had not yet been isolated, and that the outbreak was coming under control (*7*).

When the reports of a severe respiratory disease were received by WHO on February 11, 2003, a new strain of influenza virus was the most feared potential cause, and the WHO Global Influenza Network was again alerted. Concern grew on February 20, when the network received reports from Hong Kong authorities confirming the detection of A(H5N1) avian influenza virus in two persons, and WHO activated its influenza pandemic preparedness plans (*8*).

During that same week, laboratories of the WHO Global Influenza Surveillance Network began analyzing specimens from a patient with severe atypical pneumonia hospitalized in Hanoi after travel to Hong Kong. Concurrently, GOARN response teams in Vietnam and Hong Kong began collecting clinical and epidemiologic information about the patient and a growing number of others with similar symptoms. As more specimens entered the network laboratories, influenza viruses were ruled out as the causative agent. WHO made its first global alert on March 12, followed by a second, on March 15, when more than 150 suspected new cases had been reported from several geographic areas, including Hong Kong, Singapore, Vietnam and Canada (*9*,*10*). With the second alert, WHO provided a case definition and name, thus beginning a coordinated global outbreak response that brought heightened vigilance everywhere and intense control efforts. GOARN linked some of the world’s best laboratory scientists, clinicians, and epidemiologists electronically, in virtual networks that provided rapid knowledge about the causative agent, mode of transmission, and other epidemiologic features (*11*). This real-time information made it possible for WHO to provide specific guidance to health workers on clinical management and protective measures to prevent further nosocomial spread. It also made possible recommendations to international travelers to curtail international spread. Recommendations were at first nonspecific, urging international travelers to have a high level of suspicion if they had traveled to or from areas where the outbreak was occurring. But as more information became available, airports were asked to screen passengers for history of contact with SARS and for persons with current illness that fit the SARS case definition. Finally, when these recommendations did not completely stop international spread, passengers themselves were asked to avoid travel to areas where contact tracing was unable to link all cases to known chains of transmission (*12*). Within 4 months, transmission of SARS had been interrupted at all sites, and on July 5, 2003, the SARS outbreak was declared contained (*13*).

As many times occurs with emerging and reemerging infectious diseases, national surveillance mechanisms failed to identify and respond to the emerging outbreak of SARS early enough to prevent its toll of sickness, death, and international spread (*14*). In May 2003, ministers of health from the 192 member countries of WHO expressed their deep concern about the impact of SARS and its implications for future outbreaks, which were considered inevitable. In two resolutions, they called for increased national capacity development for surveillance and response and endorsed the ways in which GOARN obtained information about SARS and supported containment efforts (*15*,*16*). The resolutions stressed the need for countries to give more attention, with WHO support, to the strengthening of national surveillance and response capacity, and encouraged WHO to continue to strengthen GOARN, its safety net for global alert and response. As SARS so amply demonstrated, protection against the threat of emerging and epidemic-prone diseases requires strong defense systems at national as well as international levels.

## References

[1] Tauxe RV , Mintz ED , Quick RE . Epidemic cholera in the New World: translating field epidemiology into new prevention strategies. Emerg Infect Dis. 1995;1:141–6. 10.3201/eid0104.950408 8903186 PMC2626892

[2] Plague—international team of experts, India. Wkly Epidemiol Rec. 1994;69:321–2. 7803220

[3] Khan AS , Tshioko FK , Heymann DL , Le Guenno B , Nabeth P , Kerstiens B , The re-emergence of Ebola hemorrhagic fever, Democratic Republic of the Congo, 1995. J Infect Dis. 1999;179(suppl1):S76–86. 10.1086/514306 9988168

[4] Heymann DL , Rodier GR ; WHO Operational Support Team to the Global Outbreak Alert and Response Network. Hot spots in a wired world: WHO surveillance of emerging and re-emerging infectious diseases. Lancet Infect Dis. 2001;1:345–53. 10.1016/S1473-3099(01)00148-7 11871807

[5] SARS—Chronology of events. Ottawa: Health Canada, Population and Public Health Branch; 2003.

[6] Zhong NS , Zheng BJ , Li YM , Poon LLM , Xie ZH , Chan KH , Epidemiology and cause of severe acute respiratory syndrome (SARS) in Guangdong, People’s Republic of China, in February, 2003. Lancet. 2003;362:1353–8. 10.1016/S0140-6736(03)14630-2 14585636 PMC7112415

[7] Acute respiratory syndrome, China. Wkly Epidemiol Rec. 2003;78:41.

[8] Influenza A . (H5N1), Hong Kong Special Administrative Region of China. Wkly Epidemiol Rec. 2003;78:49–50. 12674023

[9] WHO issues a global alert about cases of atypical pneumonia. [cited December 15, 2003]. Available from: URL: http://www.who.int/csr/sars/archive/2003_03_12/en/14514253

[10] World Health Organization issues emergency travel advisory. [cited December 15, 2003]. Available from: URL: http://www.who.int/csr/sars/archive/2003_03_15/en/

[11] World Health Organization Multicentre Collaborative Network for Severe Acute Respiratory Syndrome (SARS) Diagnosis. A multicentre collaboration to investigate the cause of severe acute respiratory syndrome. Lancet. 2003;361:1730–3. 10.1016/S0140-6736(03)13376-4 12767752 PMC7119328

[12] Severe acute respiratory syndrome, update 92–chronology of travel recommendations, areas with local transmission. [cited December 15, 2003]. Available from: URL: http://www.who.int/csr/don/2003_07_01/en/12899024

[13] Severe acute respiratory syndrome, update 96–Taiwan, China: SARS transmission interrupted in last outbreak area. [cited December 15, 2003]. Available from: URL: http://www.who.int/csr/don/2003_07_01/en/

[14] Breiman RF , Evans MR , Preiser W , Maguire J , Schnur A , Li A , Role of China in the quest to define and control severe acute respiratory syndrome. Emerg Infect Dis. 2003;9:1037–41. 14519236 10.3201/eid0909.030390 PMC3016762

[15] World Health Organization. Severe acute respiratory syndrome. Geneva: World Health Organization; 2003 (World Health Assembly resolution WHA56.29. [cited December 15, 2003]. Available from: URL: http://www.who.int/gb/EB_WHA/PDF/WHA56/ea56r29.pdf

[16] World Health Organization. Revision of the international health regulations. Geneva: World Health Organization, 2003 (World Health Assembly resolution WHA56.28). [cited December 15, 2003]. Available from: URL:http://www.who.int/gb/EB_WHA/PDF/WHA56/ea56r28.pdf

